# Predictive value of pre-transplant platelet to lymphocyte ratio for hepatocellular carcinoma recurrence after liver transplantation

**DOI:** 10.1186/s12957-015-0472-2

**Published:** 2015-02-18

**Authors:** Weiliang Xia, Qinghong Ke, Ye Wang, Weilin Wang, Min Zhang, Yan Shen, Jian Wu, Xiao Xu, Shusen Zheng

**Affiliations:** Division of Hepatobiliary and Pancreatic Surgery, Department of Surgery, First Affiliated Hospital, School of Medicine, Zhejiang University, Qingchun Road 79, 310003 Hangzhou, China; Key Laboratory of Combined Multi-organ Transplantation, Ministry of Public Health, First Affiliated Hospital, School of Medicine, Zhejiang University, Qingchun Road 79, 310003 Hangzhou, China

**Keywords:** Liver transplantation, Hepatocellular carcinoma, Platelet to lymphocyte ratio

## Abstract

**Background:**

Platelet to lymphocyte ratio (PLR) is a prognostic factor for various tumors, but the current opinion on the prognostic value of PLR in liver transplantation (LT) for hepatocellular carcinoma (HCC) is still controversial. The aim of this study was to investigate the value of pre-transplant PLR for predicting post-LT HCC recurrence and further evaluate the correlation of PLR with tumor-related characteristics.

**Methods:**

The clinical data of 343 LT for HCC was retrospectively analyzed. The receiver operating characteristic (ROC) curve was used to determine the optimal PLR cut-off value to predict HCC recurrence after LT. The tumor-free survival rates were compared between high and low PLR groups divided by different pre-transplant PLR cut-off values. The relationship of elevated PLR and tumor-related characteristics were also analyzed. Additionally, the tumor-free survival was compared according to different platelet and lymphocyte counts.

**Results:**

PLR 125 was the most significant cut-off value in predicting tumor-free survival after LT, with the sensitivity and specificity of 61.6% and 62.7%, respectively. PLR ≥125 was associated with significantly higher proportion of multiple tumors, large tumor size, and micro- and macro-vascular invasion than PLR <125. Of patient with PLR <125, 46.9%, 54.2%, and 61.5% were within Milan, UCSF, and Hangzhou criteria, respectively, significantly higher than 16.4%, 18.2%, and 29.1% in the PLR ≥125 group, respectively. There was no relationship between tumor-free survival and platelet or lymphocyte count independently.

**Conclusions:**

Pre-transplant PLR ≥125 was associated with advanced tumor stage and aggressive tumor behavior, and it can be used as a prognostic factor for post-transplant HCC recurrence.

## Background

Hepatocellular carcinoma (HCC) is the fifth most common cancer worldwide and the third cause of tumor-related death [1]. Liver transplantation (LT) is an ideal option for well-selected HCC patients because this treatment completely removes not only the tumor but also the underlying cirrhotic liver disease [2]. In 1996, the Milan criteria was introduced to optimize the clinical outcome of HCC patients after LT, but it has been proven to be too strict; a large proportion of patients with HCC beyond Milan criteria also have a substantial curative chance after LT. Thereafter, several expanded criteria were introduced in clinical practice [3-6]. The University of California San Francisco (UCSF) criteria and Hangzhou criteria were presented by Yao *et al.* in 2001 [5] and by Zheng *et al.* in 2008 [6], respectively.

Most of current selection criteria are based on tumor number, tumor size, and macro-vascular invasion which are evaluated by pre-transplant radiological imaging. However, the accuracy of radiological imaging is unsatisfactory, even unacceptable [7]. On the other hand, the tumor biological behavior such as histological differentiation and micro-vascular invasion cannot be evaluated by radiological imaging, and these two factors are strongly associated with an increased risk of tumor recurrence after LT [8-10]. This situation prompts us to identify other predictors of HCC recurrence after LT.

Recently, systemic inflammation is proven to be related to poor prognosis and increased tumor progression. The tumor can upregulate the inflammatory process, and the inflammatory cells can release cytokines and mediators to promote angiogenesis, tumor proliferation, and metastasis [11,12]. The platelet to lymphocyte ratio (PLR) has been used as a marker to evaluate the systemic inflammatory responses, and PLR is shown to be a prognostic factor in various tumors [13-15]. For HCC, conflicting data exist regarding the ability of PLR of predicting prognosis of HCC patients. Lai *et al.* demonstrate that PLR is a good predictor for the risk of post-LT recurrence [16], but other studies fail to find correlation between PLR and clinical outcome of HCC patients [17,18]. To date, the current opinion on the prognostic role of PLR in LT for HCC is still controversial. We therefore conducted this study to investigate the predictive value of PLR for post-transplant tumor recurrence.

## Methods

### Patients

A total of 343 patients who received LT for HCC were enrolled in this retrospective study, and all the HCC developed in the background of liver cirrhosis which was confirmed by pathology of explant liver. The exclude criteria were (1) recipient age less than 18 years, (2) patients who died during the first month after LT, (3) recipients without adequate blood records and clinical data, and (4) patients with pre-transplant sepsis, hypersplenism, or massive gastrointestinal tract bleeding. All the LT were performed in the first affiliated hospital, School of Medicine, Zhejiang University, between January 2003 and December 2013.

Ethical approval was obtained from the Committee of Ethics in Biomedical Research of Zhejiang University and conformed to the ethical guidelines of the Declaration of Helsinki. Written informed consents were obtained from all participants.

### Study design and data collection

The blood cell testing is performed every week or necessary before LT; the PLR was calculated as the ratio of platelet count to lymphocyte count according to the blood cell testing performed within 1 month before LT; if more than one set of measurement were available for a given patient, only the minimal PLR value was used. The HCC patients were divided into high PLR and low PLR groups according to the pre-transplant PLR values.

The definite diagnosis of HCC and the tumor-related characteristics including tumor number, tumor size, macro-vascular invasion, micro-vascular invasion, and tumor cell differentiation grading were judged based on pathological findings. The judgment of histological fulfillment of Milan criteria [2], UCSF criteria [5], or Hangzhou criteria [6] for a given patients was based on pathological examination of explant livers.

The recipients’ clinical variables including age, gender, model of end-stage liver disease (MELD) score, hepatitis B virus (HBV) infection status, and transplantation type (living donor liver transplantation [LDLT] or deceased donor liver transplantation [DDLT]) were collected and evaluated. The pre-transplant treatments of HCC were also recorded: surgical resection and interventional therapies including transarterial chemoembolization (TACE), radiofrequency ablation (RFA), and percutaneous ethanol injection (PEI).

### Follow-up

All transplanted recipients were followed up; the mean follow-up period was 33.7 months, ranged from 9.5 to 132.0 months. Screening for tumor recurrence was performed by α fetoprotein (AFP) measurement and ultrasonography every month during the first 6 months and performed every 2 months during the second 6 months. In the following years, the patients received examinations every 3 to 6 months or when necessary. Plain/enhanced thoracoabdominal computed tomography was performed every 6 months or when necessary. Bone scan or positron emission tomography was carried out in case of suspected HCC recurrence.

### Statistical analysis

Data were summarized using mean with standard deviation (SD) for continuous variables and percentage for discrete variables. Student’s *t* tests and Mann–Whitney *U* test were used for comparison of continuous with normal distribution and nonparametric distribution, respectively. Chi-square test was used for categorical variables. Survival analysis was performed using the Kaplan-Meier methods and compared using the log-rank test. Receiver operating characteristic (ROC) analysis was used to determine the PLR cut-off value with most significance in predicting tumor recurrence after LT; the optimal PLR cut-off value was considered when highest Youden index (sensitivity + specificity-1) was presented. Data were analyzed using SPSS 16.0 (SPSS Inc. Chicago, CA, USA). The *P* value <0.05 was considered statistically significant.

## Results

### Clinical characteristics of 343 patients received LT for HCC

A total of 343 HCC patients including 308 (89.8%) males and 35 (10.2%) females were enrolled in this study; their mean age was 49.4 (from 19.0 to 71.0) years; the calculated MELD score before transplantation was 13.0 ± 6.0, 320 (93.3%) were HBV infected, 41 (12.0%) patients received LDLT, and 302 (88.0%) received DDLT. The mean follow-up period was 33.7 months, ranged from 9.5 to 132.0 months.

Of these patients, 144 (42.0%) patients fulfilled the Milan criteria; 166 (48.4%) and 193 (56.3%) patients were within the UCSF and Hangzhou criteria, respectively. Before LT, 52 (15.2%) patients received surgical tumor resections and 170 (49.6%) received interventional therapies.

### Tumor-free survival of patients according to different PLR cut-off values

**Figure 1:**
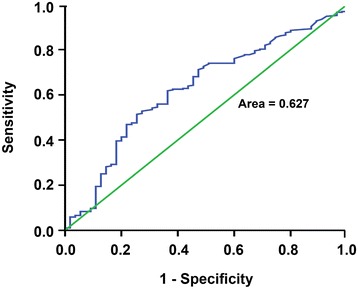
**ROC curve for the PLR values to predict HCC recurrence after LT.** The area under ROC curve was 0.627. The PLR value 125 was considered as the optimal cut-off value because of its highest Youden index; the sensitivity and specificity were 61.6% and 62.7%, respectively.

**Figure 2:**
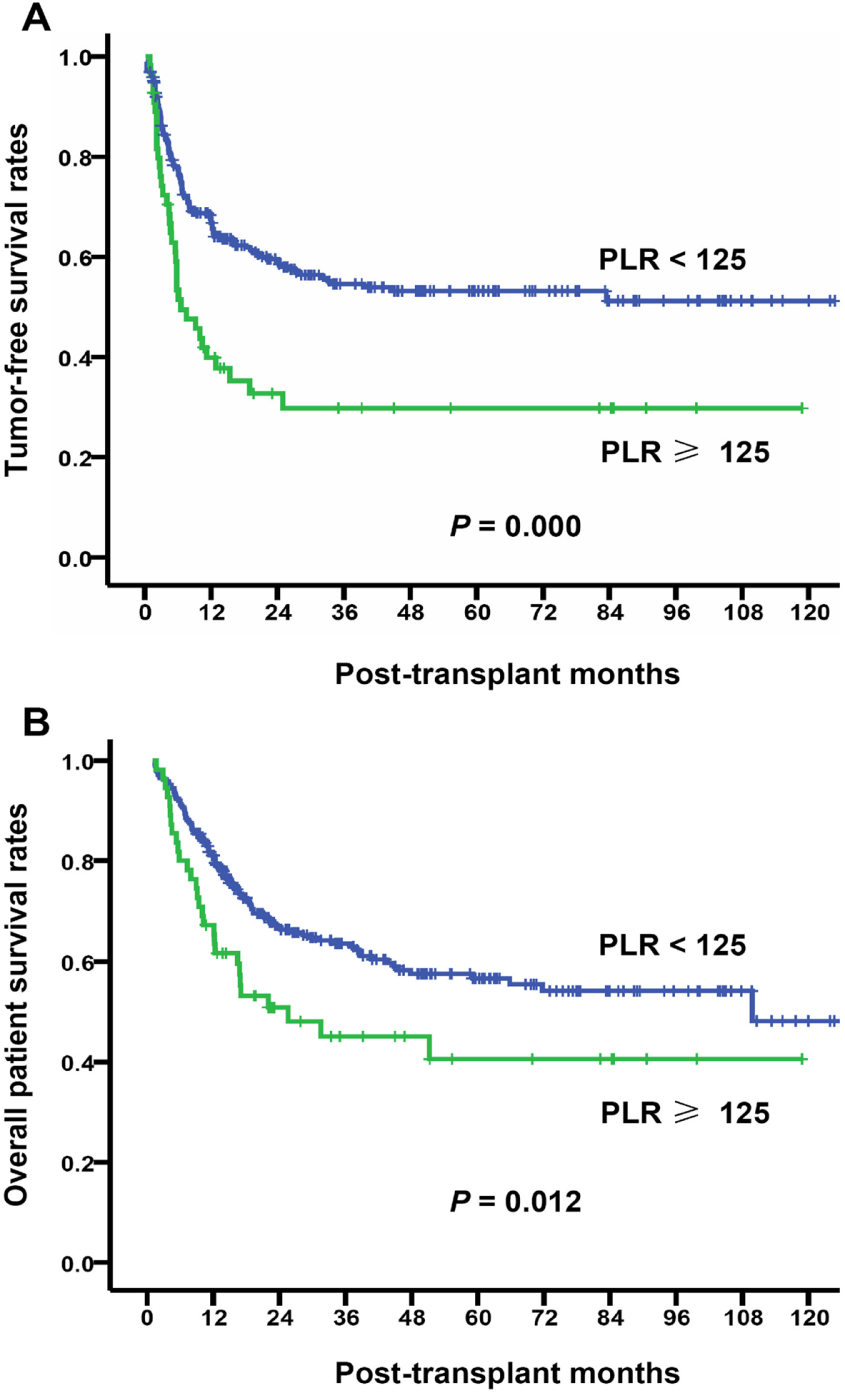
**Comparison of tumor-free survival and overall patient survival between PLR ≥125 and <125 patients.** The patients were divided into two groups according to the pre-transplant PLR cut-off value of 125. Recipients with PLR <125 presented significantly higher tumor-free survival rates (*P* = 0.000, **(A)**) and overall patient survival rates (*P* = 0.012, **(B)**) than that with PLR ≥125.

The PLR cut-off values varied among different studies ranging from 100 to 300; some studies used PLR 160 or less, while others used more than 160 [19]. As the first step, we used the ROC curve to determine the PLR cut-off value with the most significance in predicting HCC recurrence after LT. The area under ROC curve was 0.627, and the Youden index was highest when the PLR was 125; the sensitivity and specificity were 61.6% and 62.7%, respectively. Therefore, we considered PLR = 125 as the optimal cut-off (Figure 1). The 1-, 3-, and 5-year tumor-free survival rates were 66.8%, 54.6%, and 53.1% in PLR <125 patients, respectively, significantly higher than 39.9%, 29.8%, and 29.8% in PLR ≥125 patients, respectively (Figure 2A). We further compared the overall patient survival rates between PLR ≥125 and <125 patients; of patients with PLR <125, the 1-, 3-, and 5-year overall patient survival rates were 81.0%, 63.6%, and 56.7%, respectively, significantly better than 67.3%, 45.0%, and 40.5% of patients in PLR ≥125 group, respectively (*P* = 0.012, Figure 2B).

In addition, because various PLR cut-off values were used in previous studies, we also compared the tumor-free survival rates according to different values. We found that significant differences were presented when PLR cut-off values of 100, 125, and 150 were used (Table 1). Of these, the cut-off value of 125 was the most significant (*P* = 0.000). In the next analysis, we used the PLR 125 as the cut-off value.

**Table 1:** **Tumor-free survival rates of patients according to different PLR cut-off values**

**Cut-off values**	***n***	**Tumor-free survival rates (%)** ^**a**^			***P*** **value**
		**1 year**	**3 years**	**5 years**	
PLR ≥50	258	65.8 *vs.* 62.2	52.1 *vs.* 50.2	49.8 *vs.* 49.4	0.710
PLR ≥100	103	67.8 *vs.* 49.5	55.9 *vs.* 38.0	54.3 *vs.* 38.0	0.003
PLR ≥125	55	66.8 *vs.* 39.9	54.6 *vs.* 29.8	53.1 *vs.* 29.8	0.000
PLR ≥150	33	65.6 *vs.* 39.3	53.4 *vs.* 24.7	52.1 *vs.* 24.7	0.001
PLR ≥200	8	63.5 *vs.* 42.9	50.8 *vs.* 42.9	49.6 *vs.* 42.9	0.380

^a^Tumor-free survival rates in the high *vs.* low PLR group.

### Comparison of recipients’ clinical characteristics between PLR ≥125 and PLR <125 groups

Patients with PLR <125 showed better prognosis than that with PLR ≥125; the clinical backgrounds of recipients in two groups were then compared. As shown in Table 2, between PLR <125 and ≥125 groups, the clinical variables including age, gender, MELD score, HBV infection status, and transplantation type were comparable, and the pre-transplant HCC treatments including surgical resection and interventional therapies were also similar. This result indicated that the clinical characteristics of recipients in two groups were comparable.

**Table 2:** **Comparison of clinical characteristics between PLR ≥125 and PLR <125 groups**

**Variables**	**PLR**		***P*** **value**
	**<125 (** ***n*** **= 288)**	**≥125 (** ***n*** **= 55)**	
Age (years)	49.6 ± 8.6	48.5 ± 11.1	0.480
Gender (male)	260 (90.3%)	48 (87.3%)	0.500
MELD score	13.0 ± 6.0	12.7 ± 6.5	0.755
Types of LT			
LDLT	35 (12.2%)	6 (10.9%)	0.794
DDLT	253 (87.8%)	49 (89.1%)	
HBV infection	270 (93.8%)	50 (90.9%)	0.390
Pre-LT treatment			
Surgical resection	43 (17.1%)	9 (9.8%)	0.786
Interventional therapy	139 (48.3%)	31 (56.4%)	0.271

### Influences of tumor-related characteristics on tumor-free survival

We next examined that whether the different tumor recurrence was caused by disparity of tumor-related characteristics (tumor number, largest and total tumor size, macro- and micro-vascular invasion, tumor differentiation, pre-transplant AFP level and fulfill of Milan, UCSF, or Hangzhou criteria) between two groups or not. We analyzed the influences of tumor-related characteristics on tumor-free survival rates. As shown in Table 3, all of the tumor-related characteristics examined in this study were proven to be predictive for tumor-free survival after LT.

**Table 3:** **Influence of tumor-related characteristics on tumor-free survival rates**

**Tumor-related characteristics**	***n***	**Tumor-free survival rates (%)**			***P*** **value**
		**1 year**	**3 years**	**5 years**	
Tumor number					
>3	91	30.3	20.0	16.6	0.000
≤3	252	74.0	61.4	60.7	
Maximal tumor size (cm)					
≤5	233	71.9	62.1	61.2	0.000
5 to 8	54	56.4	42.1	36.9	
>8	56	27.3	11.8	11.8	
Total tumor size (cm)					
≤5	154	81.8	73.0	73.0	0.000
5 to 8	54	71.9	53.3	53.3	
>8	135	36.0	23.0	19.9	
Macro-vascular invasion					
Yes	101	38.9	18.0	18.0	0.000
No	242	71.9	63.5	61.9	
Micro-vascular invasion					
Yes	142	44.4	26.1	24.9	0.000
No	201	74.8	68.0	66.9	
Differentiation					
Poor	148	56.8	43.5	42.1	0.003
Moderate	171	63.8	52.4	51.3	
Well	24	95.8	81.2	81.2	
AFP level (ng/ml)					
≥200	178	46.9	33.5	31.4	0.000
<200	165	80.4	70.6	70.6	
Milan criteria					
Yes	144	85.7	76.7	76.7	0.000
No	199	45.0	30.5	28.1	
UCSF criteria					
Yes	166	84.3	75.6	75.6	0.000
No	177	41.5	26.2	23.6	
Hangzhou criteria					
Yes	193	83.3	74.2	74.2	0.000
No	150	35.1	20.0	17.3	

### Relationship between PLR value and tumor-related characteristics

We then evaluated the distribution of tumor-related characteristics in PLR ≥125 and <125 groups. We found that patients with PLR ≥125 displayed high proportion of multiple tumors, large tumor size, and micro- and macro-vascular invasion, but there was no difference between two groups in terms of tumor differentiation and AFP levels. For histological fulfillment of selection criteria, 46.9%, 54.2%, and 61.5% patients with PLR <125 were within Milan, UCSF, and Hangzhou criteria, respectively, significantly higher than 16.4%, 18.2%, and 29.1% in PLR ≥125 group, respectively (Table 4). This result indicated that patients with PLR ≥125 tended to be associated with multiple tumors, large tumor size, and micro- and macro-vascular invasion, and this disparity maybe the explanation of the poor prognosis of patients with PLR ≥125.

**Table 4:** **Relationship between PLR value and tumor-related characteristics**

**Variables**	**PLR**		***P*** **value**
	**<125 (** ***n*** **= 288)**	**≥125 (** ***n*** **= 55)**	
Tumor number >3	69 (24.0%)	22 (40.0%)	0.014
Largest tumor size (cm)			
≤5	214 (74.3%)	19 (34.5%)	0.000
5 to 8	43 (14.9%)	11 (20.0%)	
>8	31 (10.8%)	25 (45.5%)	
Total tumor size (cm)			
≤5	144 (50.0%)	10 (18.2%)	0.000
5 to 8	46 (16.0%)	8 (14.5%)	
>8	98 (34.0%)	37 (67.3%)	
Macro-vascular invasion	78 (27.1%)	23 (41.8%)	0.028
Micro-vascular invasion	109 (37.8%)	33 (60.0%)	0.002
Tumor differentiation			
Poor	124 (43.1%)	24 (43.6%)	0.246
Moderate	141 (49.0%)	30 (54.5%)	
Well	23 (8.0%)	1 (1.8%)	
AFP ≥200 ng/ml	136 (47.2%)	32 (58.2%)	0.147
Within Milan criteria	135 (46.9%)	9 (16.4%)	0.000
Within UCSF criteria	156 (54.2%)	10 (18.2%)	0.000
Within Hangzhou criteria	177 (61.5%)	16 (29.1%)	0.000

### Comparison of HCC recurrence according to platelet and lymphocyte counts

About the molecular mechanisms of relationship between PLR and tumor recurrence, it is hypothesized that patients with elevated PLR have a high percentage of platelet which can secrete cytokines to stimulate angiogenesis and tumor progression and low percentage of lymphocytes which play a vital role in antitumor immunological response. To check this explanation, we compared HCC recurrence according to the platelet and lymphocyte counts. The patients were divided according to the mean platelet count, those with high (≥100.3*10^9^/L, *n* = 182) and low (<100.3*10^9^/L, *n* = 161) platelet groups. The 1-, 3-, and 5-year tumor-free survival rates were 68.8%, 55.7%, and 54.6% in the low platelet group, respectively, comparable with 56.7%, 46.1%, and 44.5% in the high platelet group, respectively (*P* = 0.079, Table 5). For the lymphocyte count, patients were also divided according to the mean lymphocyte count. We found that the 1-, 3-, and 5-year tumor-free survival rates were 56.4%, 49.1%, and 47.4%, respectively, in the high lymphocyte group (≥1.1*10^9^/L, *n* = 130), similar with 69.4%, 54.2%, and 53.2%, respectively, in the low lymphocyte group (<1.1*10^9^/L, *n* = 213) (*P* = 0.168, Table 5).

**Table 5:** **Comparison of tumor-free survival according to platelet and lymphocyte counts independently**

**Groups**	***n***	**Tumor-free survival rates (%)**			***P*** **value**
		**1 year**	**3 years**	**5 years**	
Platelet count					
High (≥100.3*10^9^/L)	182	56.7	46.1	44.5	0.079
Low (<100.3*10^9^/L)	161	68.8	55.7	54.6	
Lymphocyte count					
High (≥1.1*10^9^/L)	130	56.4	49.1	47.4	0.168
Low (<1.1*10^9^/L)	213	69.4	54.2	53.2	

## Discussion

Since the induction of Milan criteria by Mazzaferro *et al.* in 1996, excellent clinical outcome after LT was achieved for HCC patients within Milan criteria [2,20]. In the following decades, the selection criteria were expanded properly without sacrificing clinical outcomes [5,6]. But most of current selection criteria are based solely on pre-transplant radiological imaging, with no consideration of the tumor biological behavior. As a result, nearly 15% ~ 20% recurrence rates of HCC after LT are reported in patients who fulfill the Milan or UCSF criteria [21,22].

On the other hand, pre-transplant radiological imaging is reported to be inaccurate [7]. In most cases, pre-transplant radiological imaging always underestimates the real tumor status because of the limitation in finding small tumor lesions. Simultaneously, radiological examination cannot evaluate the tumor differentiation and micro-vascular invasion which are strongly associated with an increased risk of tumor recurrence after LT [8-10]. Despite being within Milan or UCSF criteria, patients with these characteristics will likely response poorly to LT.

In recently years, accumulative evidences have demonstrated that increased systemic inflammation is related to poor prognosis of various kinds of cancers including colorectal cancer, pancreatic cancer, and prostate cancer [23-25]. PLR is a simple marker of systemic inflammation and can be obtained easily from routine blood cell testing. In this study, we identified that PLR ≥125 showed most significant correlation with tumor recurrence. Furthermore, we compared the clinical background and tumor-related characteristics between PLR ≥125 and <125 groups; we found that PLR ≥125 was associated with high proportion of multiple tumors, large tumor size, and micro- and macro-vascular invasion, and PLR ≥125 was prone to be beyond Milan, UCSF, and Hangzhou criteria. But we failed to find correlations of PLR with tumor histological differentiation and AFP levels. The association of PLR- and tumor-related characteristics was explored in previous studies. Kwon *et al.* showed that patients with higher PLR showed a higher likelihood of positive lymph node in colorectal cancer [26]. In cervical cancer, increased PLR was related to bigger tumor size and lymph node metastasis [27]. Azab *et al.* reported that increased PLR predicted a higher rate of lymph node involvement, higher rate of metastasis, and higher American Joint Committee on Cancer (AJCC) staging in breast cancer patients [28]. To our knowledge, it is the first study to investigate the relationship between PLR- and HCC-related characteristics, and our finding indicated elevated PLR implying high possibility of advanced tumor stage and aggressive tumor phenotype.

The molecular mechanisms involved in the relationship between PLR and tumor recurrence still remains unclear; one explanation is that patients with elevated PLR have a high percentage of platelets and low percentage of lymphocytes. The platelets can secret vascular endothelial growth factor (VEGF) which can cause angiogenesis and tumor progression [29]. The low percentage of lymphocytes indicated impaired host immunological response to malignancy [30]. But our results did not support this explanation, we failed to find differences of tumor-free survival between high and low platelet count groups or between high and low lymphocyte count groups. Recent study indicated that tumor microenvironment maybe involved in the association of systematic inflammation with tumor recurrence [31]; however, the detailed mechanisms are unclear and need further study.

## Conclusions

Our study identified that elevated pre-transplant PLR was associated with advanced tumor stage and aggressive tumor phenotype, and pre-transplant PLR can be used as a prognostic factor for post-transplant tumor recurrence.

## Abbreviations

AFP: α fetoprotein
AJCC: American Joint Committee on Cancer
DDLT: deceased donor liver transplantation
HCC: hepatocellular carcinoma
HBV: Hepatitis B virus
LT: liver transplantation
LDLT: living donor liver transplantation
MELD: model of end-stage liver disease
PEI: percutaneous ethanol injection
PLR: platelet to lymphocyte ratio
RFA: radiofrequency ablation
ROC: receiver operating characteristic
SD: standard deviation
TACE: transarterial chemoembolization
UCSF: University of California San Francisco
VEGF: vascular endothelial growth factor

## References

[1] El-Serag HB (2011). Hepatocellular carcinoma. N Engl J Med..

[2] Mazzaferro V, Regalia E, Doci R, Andreola S, Pulvirenti A, Bozzetti F (1996). Liver transplantation for the treatment of small hepatocellular carcinomas in patients with cirrhosis. N Engl J Med..

[3] Mazzaferro V, Bhoori S, Sposito C, Bongini M, Langer M, Miceli R (2011). Milan criteria in liver transplantation for hepatocellular carcinoma: an evidence-based analysis of 15 years of experience. Liver Transpl..

[4] Prasad KR, Young RS, Burra P, Zheng SS, Mazzaferro V, Moon DB (2011). Summary of candidate selection and expanded criteria for liver transplantation for hepatocellular carcinoma: a review and consensus statement. Liver Transpl..

[5] Yao FY, Ferrell L, Bass NM, Watson JJ, Bacchetti P, Venook A (2001). Liver transplantation for hepatocellular carcinoma: expansion of the tumor size limits does not adversely impact survival. Hepatology..

[6] Zheng SS, Xu X, Wu J, Chen J, Wang WL, Zhang M (2008). Liver transplantation for hepatocellular carcinoma: Hangzhou experiences. Transplantation..

[7] Freeman RB, Mithoefer A, Ruthazer R, Nguyen K, Schore A, Harper A (2006). Optimizing staging for hepatocellular carcinoma before liver transplantation: a retrospective analysis of the UNOS/OPTN database. Liver Transpl..

[8] Ciccarelli O, Lai Q, Goffette P, Finet P, De Reyck C, Roggen F (2012). Liver transplantation for hepatocellular cancer: UCL experience in 137 adult cirrhotic patients. Alpha-foetoprotein level and locoregional treatment as refined selection criteria. Transpl Int.

[9] Li WX, Li Z, Gao PJ, Gao J, Zhu JY (2014). Histological differentiation predicts post-liver transplantation survival time. Clin Res Hepatol Gastroenterol..

[10] Toso C, Asthana S, Bigam DL, Shapiro AM, Kneteman NM (2009). Reassessing selection criteria prior to liver transplantation for hepatocellular carcinoma utilizing the Scientific Registry of Transplant Recipients database. Hepatology..

[11] Coussens LM, Werb Z (2002). Inflammation and cancer. Nature..

[12] Mantovani A, Allavena P, Sica A, Balkwill F (2008). Cancer-related inflammation. Nature..

[13] Neofytou K, Smyth EC, Giakoustidis A, Khan AZ, Cunningham D, Mudan S (2014). Elevated platelet to lymphocyte ratio predicts poor prognosis after hepatectomy for liver-only colorectal metastases, and it is superior to neutrophil to lymphocyte ratio as an adverse prognostic factor. Med Oncol..

[14] Krenn-Pilko S, Langsenlehner U, Thurner EM, Stojakovic T, Pichler M, Gerger A (2014). The elevated preoperative platelet-to-lymphocyte ratio predicts poor prognosis in breast cancer patients. Br J Cancer..

[15] Kilincalp S, Coban S, Akinci H, Hamamc M, Karaahmet F, Coskun Y, Ustun Y, Simsek Z, Erarslan E, Yuksel I: Neutrophil/lymphocyte ratio, platelet/lymphocyte ratio, and mean platelet volume as potential biomarkers for early detection and monitoring of colorectal adenocarcinoma. Eur J Cancer Prev. 2014 Oct 9. [Epub ahead of print]. 10.1097/CEJ.0000000000000092 25304028

[16] Lai Q, Castro Santa E, Rico Juri JM, Pinheiro RS, Lerut J (2014). Neutrophil and platelet-to-lymphocyte ratio as new predictors of dropout and recurrence after liver transplantation for hepatocellular cancer. Transpl Int..

[17] Pinato DJ, Stebbing J, Ishizuka M, Khan SA, Wasan HS, North BV (2012). A novel and validated prognostic index in hepatocellular carcinoma: the inflammation based index (IBI). J Hepatol..

[18] Kinoshita A, Onoda H, Imai N, Iwaku A, Oishi M, Fushiya N (2012). Comparison of the prognostic value of inflammation-based prognostic scores in patients with hepatocellular carcinoma. Br J Cancer..

[19] Zhou X, Du Y, Huang Z, Xu J, Qiu T, Wang J (2014). Prognostic value of PLR in various cancers: a meta-analysis. PLoS One..

[20] Hemming AW, Cattral MS, Reed AI, Van Der Werf WJ, Greig PD, Howard RJ (2001). Liver transplantation for hepatocellular carcinoma. Ann Surg..

[21] Jonas S, Bechstein WO, Steinmuller T, Herrmann M, Radke C, Berg T (2001). Vascular invasion and histopathologic grading determine outcome after liver transplantation for hepatocellular carcinoma in cirrhosis. Hepatology..

[22] Schwartz ME, D’Amico F, Vitale A, Emre S, Cillo U (2008). Liver transplantation for hepatocellular carcinoma: Are the Milan criteria still valid?. Eur J Surg Oncol..

[23] Walsh SR, Cook EJ, Goulder F, Justin TA, Keeling NJ (2005). Neutrophil-lymphocyte ratio as a prognostic factor in colorectal cancer. J Surg Oncol..

[24] Dominguez I, Fernandez-del CC (2012). Preoperative platelet-lymphocyte ratio in resected pancreatic ductal carcinoma: is it meaningful?. Am J Surg..

[25] Wu XS, Shi LB, Li ML, Ding Q, Weng H, Wu WG (2014). Evaluation of two inflammation-based prognostic scores in patients with resectable gallbladder carcinoma. Ann Surg Oncol..

[26] Kwon HC, Kim SH, Oh SY, Lee S, Lee JH, Choi HJ (2012). Clinical significance of preoperative neutrophil-lymphocyte *versus* platelet-lymphocyte ratio in patients with operable colorectal cancer. Biomarkers..

[27] Wang D, Wu M, Feng FZ, Huang HF, Yang JX, Shen K (2013). Pretreatment neutrophil-to-lymphocyte and platelet-to-lymphocyte ratios do not predict survival in patients with cervical cancer treated with neoadjuvant chemotherapy and radical hysterectomy. Chin Med J (Engl)..

[28] Azab B, Shah N, Radbel J, Tan P, Bhatt V, Vonfrolio S (2013). Pretreatment neutrophil/lymphocyte ratio is superior to platelet/lymphocyte ratio as a predictor of long-term mortality in breast cancer patients. Med Oncol..

[29] Bambace NM, Holmes CE (2011). The platelet contribution to cancer progression. J Thromb Haemost..

[30] Dunn GP, Old LJ, Schreiber RD (2004). The immunobiology of cancer immunosurveillance and immunoediting. Immunity..

[31] Motomura T, Shirabe K, Mano Y, Muto J, Toshima T, Umemoto Y (2013). Neutrophil-lymphocyte ratio reflects hepatocellular carcinoma recurrence after liver transplantation *via* inflammatory microenvironment. J Hepatol..

